# Mycorrhizal symbiosis and application of vitamin B3-treated *Trichoderma Harzianum* HE24 additively trigger immunity responses in faba bean plants against Rhizoctonia root rot and promote the plant growth and yield

**DOI:** 10.1186/s12870-025-06950-8

**Published:** 2025-07-19

**Authors:** Hany H.A. El-Sharkawy, Younes M. Rashad, Salama S. A. El-Blasy, Basma H. Amin, Mahmoud A.A. Youssef, Mohamed Hafez, Ahmed Abd-ElGawad, Mohamed Bourouah, Elsherbiny A. Elsherbiny, Safaa A. Yousef

**Affiliations:** 1 Mycology Research and Plant Diseases Survey Department, Plant Pathology Research Institute, Agricultural Research Center, Giza, Egypt; 2 Plant Protection and Biomolecular Diagnosis Department, Arid Lands Cultivation Research Institute (ALCRI), City of Scientific Research and Technological Applications (SRTA-City), New Borg El-Arab, Alexandria, 21934 Egypt; 3 Leguminous and Forage Crop Diseases Department, Plant Pathology Research Institute, Agricultural Research Center, Giza, Egypt; 4 The Regional Center for Mycology and Biotechnology (RCMB), Al-Azhar University, Cairo, Egypt; 5 Seed Pathology Research Department, Plant Pathology Research Institute, Agricultural Research Center, Giza, Egypt; 6 Land and Water Technologies Department, Arid Lands Cultivation Research Institute (ALCRI), City of Scientific Research and Technological Applications (SRTA-City), New Borg El-Arab, 21934 Egypt; 7 Plant Production Department, College of Food & Agriculture Sciences, King Saud University, P.O. Box 2460, Riyadh, 11451 Saudi Arabia; 8 Hahn-Schickard-Gesellschaft für Angewandte Forschung e.V, Wilhelm- Schickard-Straße, Villingen-Schwenningen, 1078052 Germany; 9 Plant Pathology Department, Faculty of Agriculture, Mansoura University, Mansoura, Egypt

**Keywords:** Biocontrol, Vicia faba, Vitamin B3, Arbuscular mycorrhizal Fungi, *Rhizoctonia Solani*

## Abstract

**Background:**

Faba bean is a valuable legume crop, known for its high nutritional value and nitrogen fixing ability. Rhizoctonia root rot of faba bean, caused by *Rhizoctonia solani*, severely affects the plant growth and yield. This study aimed to evaluate the effect of symbiosis with arbuscular mycorrhizal fungi (AMF) and application of vitamin B3-treated *Trichoderma harzianum* HE24 on plant immune responses against Rhizoctonia root rot under greenhouse conditions.

**Results:**

Results revealed that symbiosis with AMF and the application of vitamin B3-treated *T. harzianum* HE24 significantly upregulated the defense-related genes *CHI II* (50.2-fold), *PAL1* (13.3-fold), and *HQT* (37.0-fold). Additionally, this combined treatment led to an increment in the enzymatic activity of peroxidase and polyphenol oxidase recording 23.7 and 14.6-unit min^−1^g^−1^ fresh weight, respectivly. Furthermore, the phenolic content in faba bean plants was enhanced (1402.3 mg/g fresh weight) suggesting a strong activation of the plant’s biochemical defense mechanisms and metabolic activities. Rhizoctonia root rot severity in faba bean plants was reduced by 80.4% following this treatment. Moreover, the results demonstrated that AMF symbiosis notably improved plant growth, photosynthetic content, and yield, compared to the infected control, leading to enhanced overall plant performance and disease resistance.

**Conclusions:**

These findings suggest that the additive interaction between AMF and vitamin B3-treated *T. harzianum* HE24 provides an effective, eco-friendly alternative for managing root rot of faba bean.

## Background

Faba bean (*Vicia faba* L.) is a valuable legume crop, known for its high nutritional value and its significant role in sustainable agriculture. In Egypt, approximately 17,000 ha were cultivated with faba bean in 2023, yielding a production of 172,000 tons [1]. Its ability to fix atmospheric nitrogen enriches the soil, benefits subsequent crops and reduces dependence on synthetic fertilizers. However, faba bean is vulnerable to biotic stress caused by destructive soil-borne pathogens, such as *Rhizoctonia solani* J.G. Kühn (Rs). This aggressive pathogen infects a wide range of plant species, both monocots and dicots, causing diseases such as damping-off, seed decay, root rot, and crown rot, all of which negatively affect plant growth and yield [2, 3]. Rs is highly resilient in soil, capable of surviving for up to 20 years in the form of sclerotia. Its high genetic diversity and the low levels of innate immunity in plants make it particularly difficult to control using conventional agricultural methods [4].

Recent advances in biological control have introduced promising and sustainable solutions for managing Rs infections. Among the most effective biocontrol agents are species of the genus *Trichoderma*, fungi known for their strong antagonistic properties. *Trichoderma* spp. inhibit pathogens through resource competition, mycoparasitism, and the production of antifungal metabolites that actively suppress various plant pathogens [2]. Recent studies have shown that *Trichoderma* species can effectively reduce the growth and infection potential of Rs, offering a compelling alternative to the chemical pesticides. Wang and Zhuang [5] reported the high biocontrol potential of *T. guizhouense* 9185 and *T. simmonsii* 8702 against root rot of cowpea, achieving reductions in disease severity of 36.6 and 45.0%, respectively. In recent years, research has also explored the role of vitamins in enhancing the efficacy of beneficial microorganisms used in biological control. Vitamin B3 (niacin), in particular, has been found to stimulate the growth and metabolic activity of *Trichoderma* spp., enhancing their production of hydrolytic enzymes and antifungal compounds [4]. This supplementation not only boosts plant resistance to pathogens by promoting the synthesis of secondary metabolites, but also improves the plant’s overall defense response to fungal attacks.

Arbuscular mycorrhizal fungi (AMF) are biotrophic fungi that symbiotically colonize the roots of more than 80% of terrestrial plant species forming a mutualistic relationship in which both partners benefits. This association results in healthier and more vigorous plant growth. AMF have been widely and successfully studied as biocontrol agents against numerous fungal, bacterial, and viral plant diseases [3, 6]. The ability to induce plant immunity at the biochemical, molecular, and ultrastructural levels in response to invading pathogens has also been reported [7, 8]. Abdel-Fattah et al. [9] observed several ultrastructural defense responses in common bean plants colonized by AMF when infected with Rhizoctonia root rot. These included cell wall lignification, cytoplasmic granulation, and an increment in total phenolic content. In addition, AMF enhance plant health by improving nutrient uptake, particularly phosphorus, and promoting root development [2].

The integration of AMF with vitamin B3-treated *T. harzianum* may offer a comprehensive and sustainable biocontrol strategy. This study aimed to evaluate the antifungal efficacy of vitamin B3-treated *T. harzianum* HE24 against Rs, and to investigate its interaction with AMF in managing Rhizoctonia root rot of faba bean. Additionally, the study examined the effects of these treatments on plant growth, yield, and the associated biochemical and genetic responses, contributing to the development of an eco-friendly approach to disease management.

## Methods

### Source of used fungi, chemicals, and faba bean cultivar

Seeds of the faba bean cultivar Giza 843 were obtained from the Field Research Institute, Agricultural Research Center, Giza, Egypt. Niacin, used as a supplement in this study, was purchased from Sigma Co. USA. A highly virulent isolate of Rs (AG-4), originally isolated from rotted roots of faba bean plants, was kindly provided by the Plant Pathology Research Institute, Agricultural Research Center, Egypt. A mixed inoculum of AMF, with a colonization index of 81%, was also provided by the same institute. The inoculum included spores, in equal proportions, of the following species: *Rhizophagus irregularis* (Blaszk., Wubet, Renker, and Buscot) Walker & Schüßler, *Funneliformis mosseae* (Nicolson & Gerd.) Walker & Schüßler, *Rhizoglomus clarum* (Nicolson & Schenck) Sieverd., Silva & Oehl, *Gigaspora margarita* (Becker & Hall), and *G. gigantea* (Nicol. & Gerd.) Gerd. & Trappe.

### Dual culture assay

The antagonistic effect of *T. harzianum* HE24 (with or without vitamin B3 treatment) against Rs was assessed in vitro using the dual culture technique, as described by Kucuk and Kivanc [10]. A 5 mm mycelial disc from a 5-day-old culture of *T. harzianum* HE24 (treated or not with vitamin B3) was placed on one side of a 9 cm diameter Petri dish containing potato dextrose agar (PDA). For the vitamin B3 treatment, a set of PDA plates was supplanted with 0.05% vitamin B3 prior to solidification [11]. A similar-sized mycelial disc of Rs was placed on the opposite side of the plate. The plates were incubated at 25 ± 2˚C. Each treatment was replicated three times, and the entire experiment was repeated twice to ensure consistency. Once the mycelial growth of Rs in the control plates reached the edges, the radial growth of Rs in all plates was measured. The percentage of mycelial growth inhibition was then calculated.

### Greenhouse experiment

The greenhouse experiment aimed to evaluate the biocontrol efficacy of AMF and *T. harzianum* HE24 (with or without vitamin B3 treatment), applied as seed treatments, individually and in combination, against Rhizoctonia root rot of faba bean.

### AMF and *R. solani* inoculum

The AMF inoculum was a mixture of *G. margarita*, *F. mosseae*, *R. irregularis*, and *R. clarum* in equal proportions. According to El-Sharkawy [12], 50 g of AMF inoculum was added to each pot. A spore suspension of *T. harzianum* HE24 (treated or untreatedwith vitamin B3), at 3 × 10⁶ spore mL^−1^, was prepared from 5-day-old cultures grown on PDA, following the method described by Sallam et al. [13]. Rs was cultured on PDA plates for 7 days at 25°C, after which mycelial discs were transferred to a sterilized sorghum-sand medium (2:1 v/v) and incubated for 10 days at 25 ± 2°C.

Pots (20 cm in diameter) were filled with sterilized soil composed of a sand: clay mixture (1:1 v/v) and planted with faba bean seeds (*cv.* Giza 843), 5 seeds per pot. Soil sterilization was performed using two cycles of steaming for 20 min at 120℃. The soil properties were as follows: pH 8.1, electrical conductivity 1.62 dS m^−1^, organic matter 9.1 g kg^−1^, total nitrogen 0. 38 g kg^−1^, available phosphorus 4.98 mg kg^−1^, and available potassium 345 mg kg^−1^. Initially, seeds of faba bean were surface-sterilized with 0.05% sodium hypochlorite for 2 min, followed by rinsing with sterile water. Before planting, the seeds were soaked individually for 30 min in a spore suspension of *T. harzianum* HE24 (with or without vitamin B3), supplemented with 1% carboxymethyl cellulose. Seeds soaked in sterile distilled water served as the negative control, while seeds treated with the fungicide Topsin (3 g L^−1^) served as the positive control.

For soil infestation, Rs inoculum was mixed into the potting soil at 3%. Each treatment was replicated in six pots. All pots were irrigated as needed and maintained under greenhouse conditions at 25/18°C (day/night) with 60% relative humidity. the experiment was arranged in completely randomized design with the following thirteen treatments:

1. Untreated and non-infected (C).
2. Untreated and infected (Rs).
3. Treated with chemical fungicide and infected (F + Rs).
4. Treated with *T. harzianum* HE24 and non-infected (TH).
5. Treated with *T. harzianum* HE24 + vitamin B3 and non-infected (THB).
6. Inoculated with AMF and non-infected (AMF).
7. Inoculated with AMF and treated with *T. harzianum* HE24 and non-infected (TH + AMF).
8. Inoculated with AMF and treated with *T. harzianum* HE24 + vitamin B3 and non-infected (THB + AMF).
9. Treated with *T. harzianum* HE24 and infected (TH + Rs).
10. Treated with *T. harzianum* HE24 + vitamin B3 and infected (THB + Rs).
11. Inoculated with AMF and infected (AMF + Rs).
12. Inoculated with AMF and treated with *T. harzianum* HE24 and infected (TH + AMF + Rs).
13. Inoculated with AMF and treated with *T. harzianum* HE24 + vitamin B3 and infected (TH + AMF + Rs).

### Disease assessment

Root rot severity was visually evaluated 45 days after inoculation (dai) using a 0–4 scale described by [14] as follows: 0 = no symptoms, 1 = mild necrotic lesions, 2 = necrotic lesions around the main root, 3 = necrotic lesions spreading into the root system, and affecting the root tip, and 4 = most of the root system infected, with only a small portion of uninfected tissue remain.

### Expression profiling

Faba bean plants from each treatment were uprooted and washed with tap water 14 dai. Total RNA was extracted from the sampled plants using RNeasy Mini Kit (Qiagen, Germany) following the manufacturer’s instructions. Complementary DNA (cDNA) was synthesized using a reverse transcription kit (Qiagen, Germany) and a thermal cycler (Promega, Germany). The 20 µL mixture reaction contained: 5 µL of 5X-buffer supplemented with MgCl_2_, 2.5 mM, 2.5 µL of dNTPs, 4 µL of oligo dT primer (20 pmol µL^−1^), 2 µL of RNA extract, and 0.2 µL of reverse transcriptase. The reverse transcription was performed under the following conditions:2 h at 42°C, followed by 20 min at 65°C. For quantitative Real-Time PCR (qPCR), the 20 µL reaction mixture contained 12.5 µL of SYBR Green Master Mix (Bioline, Germany), 1 µL of cDNA, 1 µL of each of the forward and reverse primers, and sterile RNase-free H_2_O to reach the final volume. The β-actin gene was utilized as a reference. The primers used in this study are listed in Table 1.

**Table 1:** Primers sequence used in the expression profiling

Gene name	Abbreviation	Sequence (5′−3′)
Chitinase class II	*CHI ΙΙ*-F *CHI ΙΙ*-R	GCGTTGTGGTTCTGGATGACA CAGCGGCAGAATCAGCAACA
Phenylalanine ammonia lyase 1	*PAL1*-F *PAL1*-R	ACGGGTTGCCATCTAATCTGACA CGAGCAATAAGAAGCCATCGCAAT
Hydroxycinnamoyl-CoA quinate hydroxycinnamoyl transferase	*HQT*-F *HQT*-R	CCCAATGGCTGGAAGATTAGCTA CATGAATCATTTCAGCCTCAACAA
*β*-actin	*β*-actin-F *β*-actin-R	GTGGGCCGCTCTAGGCACCAA
		CTCTTTGATGTCACGCACGATTTC

The qPCR reaction was performed as follows: one cycle (95°C/10 min) and 40 cycles (95°C/20 s, 58°C/25 s, and 72°C/30 s) using Rotor-Gene-6000-system (Qiagen, United States). Three biological and three technical replicates were applied for each sample. Analysis of the relative expression levels was calculated using the comparative CT method (2^−ΔΔCT^) [15].

### Biochemical analyses

Root samples were collected 15 dai to assess phenolic content and activities of peroxidase (POD) and polyphenol oxidase (PPO). POD activity was measured according to the method described by Maxwell and Bateman [16], while PPO activity was estimated following the protocol of Galeazzi et al. [17]. Total phenolic content was quantified using the Folin-Ciocalteu reagent, based on the method of Malik and Singh [18]. Total soluble solids in faba bean seeds were estimated using a hand refractometer (Master T, ATAGO Co., Japan). Additionally, photosynthetic pigments in faba bean leaves were analyzed 40 dai following the method of Lichtenthaler and Wellburn [19].

#### Mycorrhization assessment

Five faba bean roots for each treatment were assessed 30 dpi to determine the colonization status. The sampled roots were cut into 1 cm segments, and 40 segments per treatment were boiled in 10% KOH solution. The samples were then stained with trypan blue (Sigma, USA). Mycorrhizal colonization was evaluated using light microscopy. Colonization frequency, colonization intensity, and arbuscule frequency were assessed according to the method described by Trouvelot et al. [20].

### Growth and yield evaluation

At 50 days after inoculation, ten plants per treatment were sampled, carefully washed, and evaluated for the leaf area (cm²), shoot height (cm), root length (cm), and shoot and roots dry weights (g). At full maturity, another set of ten plants from each treatment were collected for yield evaluation. Yield parameters included yield per plant (kg), weight of ten pods (g), and weight of 100 seeds (g). All samples were oven-dried at 80°C to constant weight before the weighting.

### Statistical analysis

Data were analyzed using CoStat 2005 version 6.4 (CoHort Software, USA), following the method described by Duncan [21]. Treatment means were compared using Duncan’s multiple range test at a significance level of 𝑃 ≤ 0.05.

## Results

### Dual culture test

**Fig. 1:**
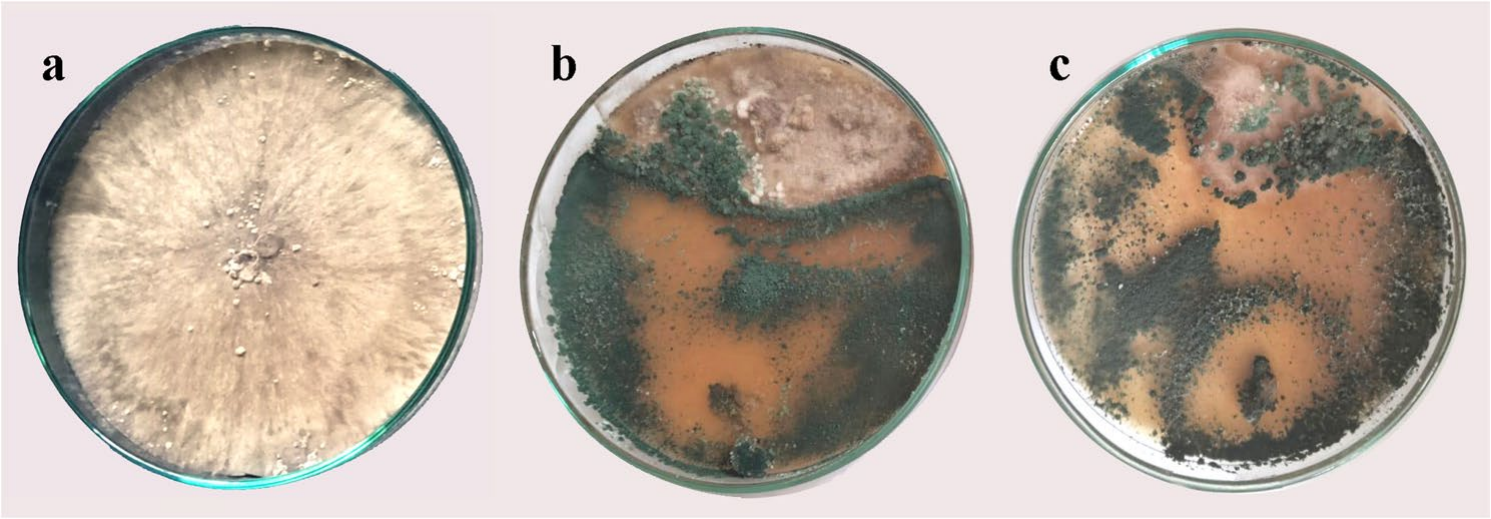
Antagonistic effects of *T. harzianum* HE24 and *Rhzoctonia solani* using the dual culture test in vitro, where **a** control culture of *R. solani*, **b** dual culture of *T. harzianum* HE24 and *R. solani*, and **c** dual culture of *T. harzianum* HE24/vitamin B3 and *R. solani*

Table 2; Fig. 1 illustrate the antagonistic effect of *T. harzianum* HE24 (with or without vitamin B3 treatment) against Rs. In the control treatment, Rs exhibited normal radial growth (9 mm, 0% inhibition), with a dense, uniform colony appearance (Fig. 1a). Dual culturing with *T. harzianum* HE24 alone significantly reduced the redial growth of Rs to 4.7 mm, achieving 48.3% inhibition (Fig. 1b). The most pronounced reduction in growth of Rs was observed when co-cultured with vitamin B3-treated *T. harzianum* HE24, where growth was drastically suppressed to 0.8 mm, recording 91.7% inhibition (Fig. 1c). Overgrowth of *T. harzianum* HE24 (treated or not with vitamin B3) over Rs colonies was also observed.

**Table 2:** Antagonistic effect of *Trichoderma harzianum* HE24 (treated or not with vitamin B3) on radial growth of *Rhizoctonia solani* in vitro

Treatment	Radial growth of *R*. *solani* (mm)	Growth inhibition (%)
Rs	9.0 ± 0.9^a^	0.0^c^
TH + Rs	4.7 ± 0.4^b^	48.3 ± 1.2^b^
THB + Rs	0.8 ± 0.5^c^	91.7 ± 2.3^a^

Means within each column followed by different letter are significantly different, as determined by Duncan’s multiple range test at *P* ≤ 0.05. Where, Rs: a single culture of *R. solani*, TH + Rs: dual culture plate containing *R. solani* and *T. harzianum* HE24, and THB + Rs: a dual culture plate containing *R. solani* and *T. harzianum* HE24 treated with vitamin B3

### SEM observations

**Fig. 2:**
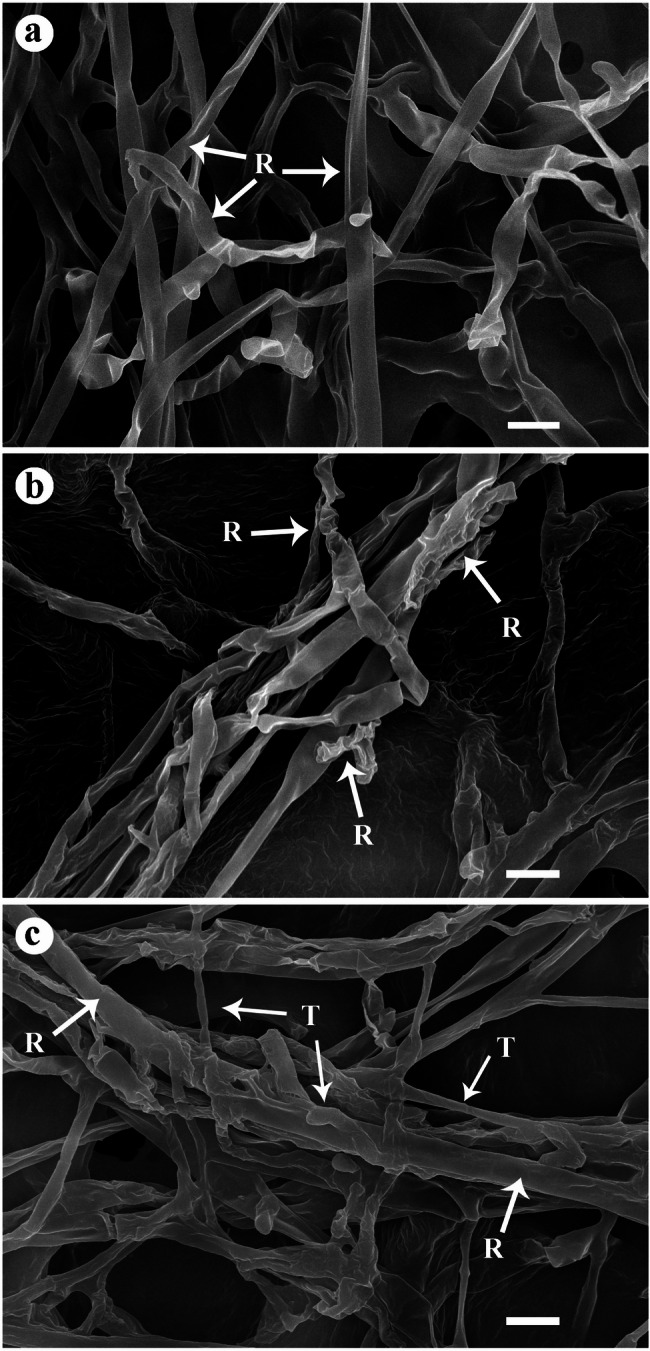
Scanning electron micrographs show interaction between mycelia of *T. harzianum* HE24 (treated or not with vitamin B3) and those of *Rhzoctonia solani* in dual culture plates. Where, **a** thick mycelia of *R. solani* exhibiting normal thick mycelial growth with branching at characteristic 90° angles, **b** dual culture of *T. harzianum* HE24 and *R. solani* showing distortion and disintegration of the pathogen mycelia in response to the active metabolites of the antagonistic *T. harzianum* HE24, and **c** dual culture of *T. harzianum* HE24/vitamin B3 and *R. solani* showing clear signs of mycoparasitism, with producing numerous swollen hyphal tips of *T. harzianum* HE24 that facilitated robust penetration and degradation of the pathogen’s mycelia (thin mycelia) on *R. solani* (thick mycelia)

SEM observations revealed significant morphological alterations in mycelia of Rs when co-cultured with *T. harzianum* HE24 (with or without vitamin B3). Mycelia of the control culture of Rs exhibited normal, thick mycelial growth with characteristic 90° branching angles. Hyphal tips often swelled into globose structures, forming appressoria, indicative of its infection mechanism (Fig. 2a). In contrast, when cultured with *T. harzianum* HE24, clear signs of antagonistic activity were observed, including disintegration and distortion of the Rs mycelia in response to the active metabolites produced by *T. harzianum* HE24 (Fig. 2b). Micrographs of Rs and *T. harzianum* HE24/vitamin B3 showed coiling of *T. harzianum* HE24 (thin mycelia) on Rs mycelia (thick mycelia) and direct hyphal penetration into the filaments of Rs (Fig. 2c). This interaction displayed distinct features of mycoparasitism, with the formation of numerous swollen hyphal tips of *T. harzianum* HE24 that facilitated robust penetration and degradation of the pathogen’s mycelia.

### Effect of applying *T. harzianum* HE24 and/or AMF on the disease severity

Table 3 demonstrates effect of treating faba bean plants with AMF and/or *T.harzianum* HE24 (treated or not with vitamin B3), either singly or in combination, on the disease severity caused by *Rs* under greenhouse conditions. Single applying of *T. harzianum* HE24 (treated or not with vitamin B3) and AMF showed a notable reduction in the disease severity of the infected plants, compared to the untreated-infected plants. Combined treatments of AMF and *T. harzianum HE24 (*treated or not with vitamin B3*)* were more effective in reducing the disease severity than the single treatments. The highest disease reduction achieved due to the tested treatments was recorded for the combined treatment (THB + AMF + Rs) recording 80% reduction, compared to the untreated-infected plants. The chemical fungicide effectively reduced disease severity recording 87% reduction in the disease severity.

**Table 3:** Effect of inoculation with arbuscular mycorrhizal fungi and/or *Trichoderma harzianum* HE24 (treated or not with vitamin B3) on disease severity of faba bean plants (*cv.* Giza 843) infected with *Rhizoctonia solani* under greenhouse conditions at 45 days after inoculation

Treatment	Disease severity	Reduction %
Rs	76.7 ± 2.7^a^	0.0^f^
TH + Rs	35.0 ± 1.8^bc^	54.4 ± 2.5^de^
THB + Rs	30.0 ± 2.4^c^	60.9 ± 2.0^d^
AMF + Rs	40.0 ± 3.1^b^	47.9 ± 2.0^e^
F + Rs	10.0 ± 2.0^e^	87.0 ± 4.6^a^
TH + AMF + Rs	21.7 ± 2.5^d^	71.7 ± 2.3^c^
THB + AMF + Rs	15.0 ± 1.9^de^	80.4 ± 3.8^ab^

Means within each column followed by different letter are significantly different, as determined by Duncan’s multiple range test at *P* ≤ 0.05. Where, Rs: untreated and infected, TH + Rs: treated with *T. harzianum* HE24 and infected, THB + Rs: treated with *T. harzianum* HE24/vitamin B3 and infected, AMF + Rs: inoculated with AMF and infected, F + Rs: treated with the chemical fungicide and infected, TH + AMF + Rs: inoculated with AMF and treated with *T. harzianum* HE24 and infected, and TH + AMF + Rs: inoculated with AMF and treated with *T. harzianum* HE24/vitamin B3 and infected

### Microscopic examination of the semi-thin sectioning

**Fig. 3:**
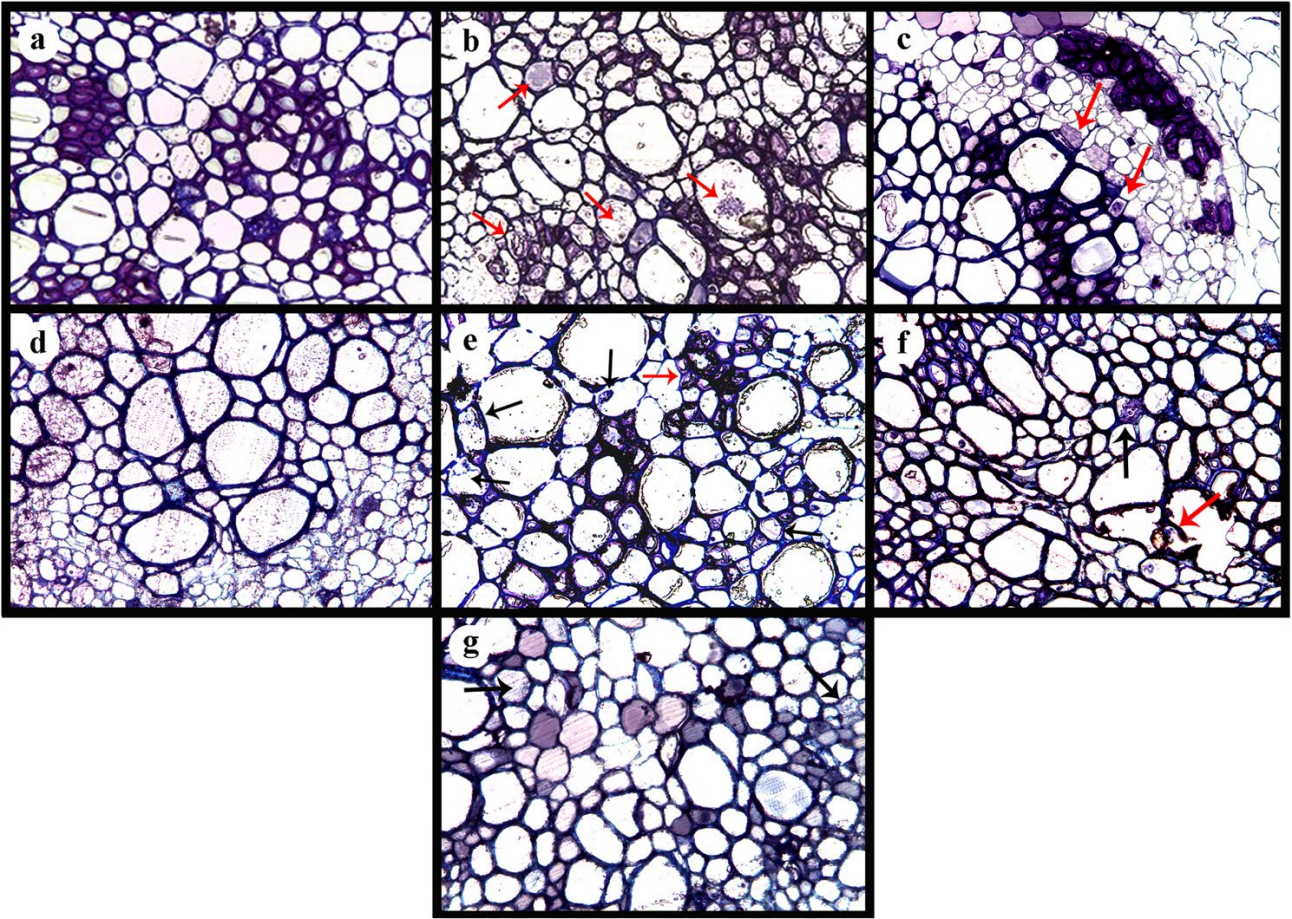
Semithin sections light microscopic images of faba bean root. **A** Healthy control (non-infected) showing a classical cellular organization of the epidermal and cortical cells, **B** infected with *R. solani* (red arrows) showing formation of specialized infection cushions, facilitating penetration into multiple epidermal cells, **C** treated with *T. harzianum* HE24 against *R. solani* (red arrow), **D** treated with *T. harziaum* HE24*/*vitamin B3 against *R. solani*, **E** treated with (AMF) Arbuscular Mycorrhizal Fungi (black arrows) against *R. solani* (red arrow), **F** treated with *T. harzianum* HE24 and AMF (black arrow) against *R. solani* (red arrow), **G** treated with *T. harziaum/*vitamin B3 and AMF (black arrows) against *R. solani.* The applied treatments showed maintained epidermal cells, with minimal pathogen intrusion, and intact vascular bundles

Microscopic examination of semi-thin sections from the stained roots of faba bean treated with various interventions revealed distinct histological features when stained with toluidine blue. In the untreated control roots, a classical cellular organization of the epidermal and cortical cells was observed, highlighting the integrity of the vascular bundle and cellular structure (Fig. 3a). In contrast, roots inoculated with Rs showed significant hyphal penetration of epidermal cells. The fungal invasion was characterized by the formation of specialized infection cushions, facilitating penetration into multiple epidermal cells (Fig. 3b). Remarkably, treatments incorporating TH (whether with or without vitamin B3) or AMF, resulted in a notable reduction in the pathogen penetration. In these treatments, the integrity of the epidermal cells was largely maintained, with only minimal pathogen intrusion observed. Moreover, the vascular bundles appeared intact, suggesting a strong defensive response elicited by these biocontrol agents (Figs. 3c–e). The THB + AMF + Rs treatment reduced the tissue damaging in the infected faba bean plants due to the infection (Fig. 3f). Among the combined treatments, THB + AMF + Rs exhibited the most significant reduction in the pathogen progression. Root tissues treated with these combinations demonstrated enhanced structural integrity, resembling the cellular architecture of the uninfected tissues. This highlights the additive effect of these biocontrol measures in suppressing fungal invasion and protecting root tissues from extensive damage (Fig. 3g).

### Effect of *T. harzianum* HE24 and/or AMF on expression of defense-related genes

**Fig. 4:**
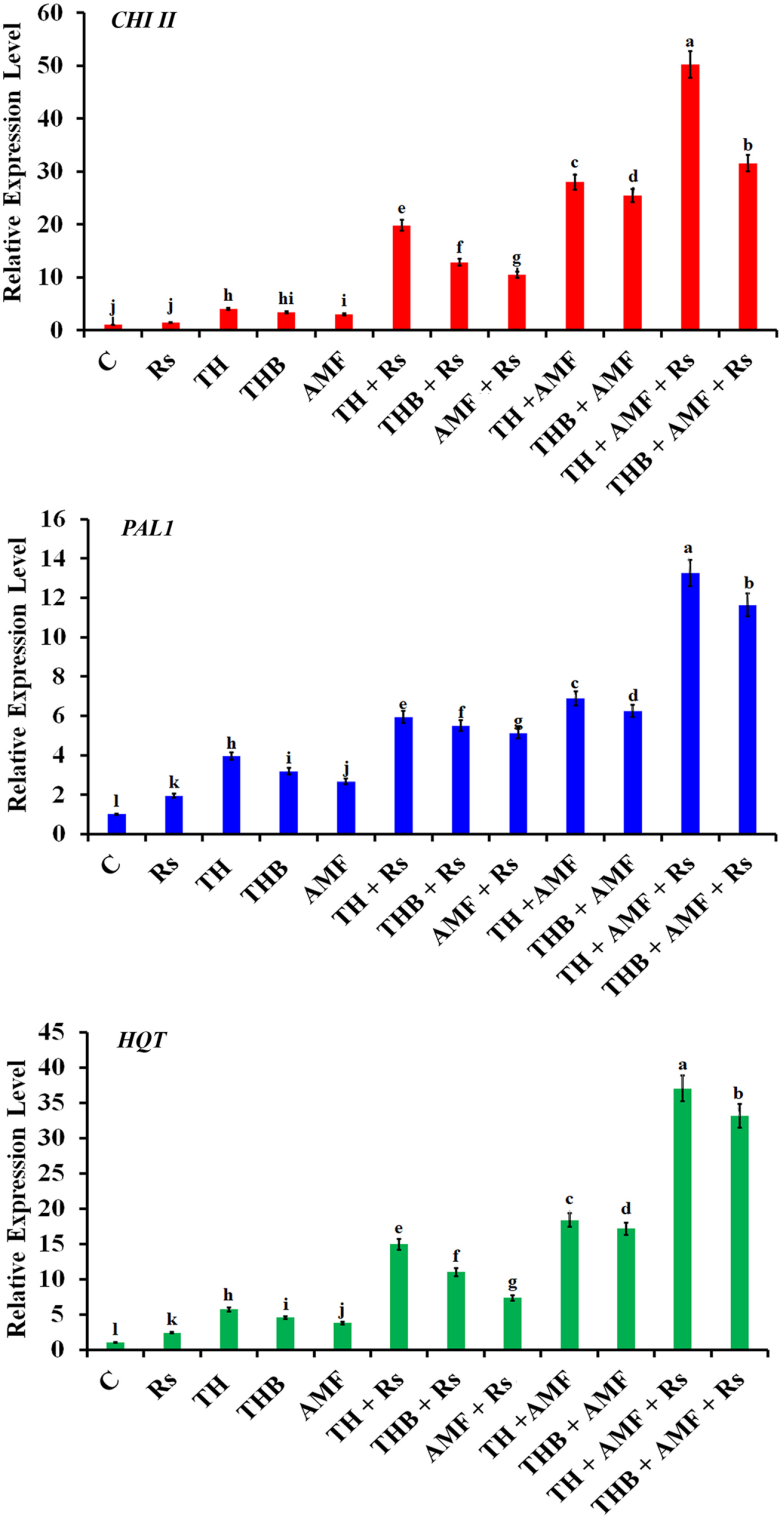
Bar chart shows effect of vitamin B3-amended *Trichoderma harzianum* HE24 and arbuscular mycorrhizal fungi on transcriptional expression level of the defense-related genes *CHI II*, *PAL1*, and *HQT* in faba bean plants in response to infection with Rhizoctonia root rot

Relative expression level of the defense-related genes (*CHI II*, *PAL1*, and *HQT*) in faba bean plants in response to the tested treatments is illustrated in Fig. 4. Results obtained revealed that infection of faba bean plants with Rs did not affect the expression level of *CHI II*, when compared with the untreated control plants. In contrast, regardless of incidence of the infection, all tested treatments of *T. harzianum* HE24 (treated or not with vitamin B3) and AMF, either alone or in combination, significantly upregulated *CHI II* to varying extent, compared to the untreated control plants. However, the upregulating effect was higher in the infected faba bean plants which were treated with any treatment of *T. harzianum* HE24 (treated or not with vitamin B3) and/or AMF, compared to the non-infected and treated plants. In this regard, the infected faba bean plants which were treated with TH recorded higher overexpressing effect than those which were treated with AMF. The highest expression level was observed for the infected faba bean plants which were treated with TH + AMF recording 50.2-fold followed by the infected faba bean plants which were treated with THB + AMF recording 31.6-fold. Regard to *PAL1*, results showed that infection of faba bean plants with Rs upregulated its expression (2-fold), compared with the untreated control plants. In the same time, all tested treatments considerably induced *PAL1* expression to varying degrees. However, the dual treatments of *T. harzianum* HE24 (treated or not with vitamin B3) and AMF recorded higher effect than the single ones. The highest expression value was recorded for the infected faba bean plants which were treated with TH + AMF recording 13.3-fold followed with the infected faba bean plants which were treated with THB + AMF recording 11.6-fold. Infection of faba bean plants with Rs led to upregulation of *HQT* by 2.4-fold, compared with the untreated control plants. All applied treatments of *T. harzianum* HE24 (treated or not with vitamin B3) and/or AMF overexpressed *HQT* with superiority of the combined treatments over the single ones, and all infected treatments over the non-infected treatments. The highest expression level was recorded for the infected faba bean plants which were treated with TH + AMF recording 37.0-fold followed with the infected faba bean plants which were treated with THB + AMF recording 33.1-fold.

### Effect of applying *T. harzianum* HE24 and/or AMF on biochemical changes

Table 4 presents effect of different treatments on some physiological defense markers (phenolic content, activity of POD and PPO, and SSC in faba bean plants. Infection with Rs significantly induced the phenolic content and enzyme activities of POD and PPO as a stress response, compared with the untreated-not infected control plants. All applied treatments of AMF and/or *T. harzianum* HE24, singly or in combinations, led to an increment in the total phenolic content, activity of POD and PPO, and SSC, compared to the negative and positive controls. However, the combined three treatments were more effective than the dual treatments and the single ones. The highest values were recorded for the treatment (THB + AMF + Rs) recording a content of total phenols of 1402.3 mg g^−1^ fresh weight and enzyme activity of POD and PPO 23.7 and 14.6 U g^−1^ fresh weight, respectively. While, the highest value of SSC was recorded for the treatment THB + AMF (23.0 °Brix).

**Table 4:** Effect of inoculation with arbuscular mycorrhizal fungi and/or *Trichoderma harzianum* HE24 (treated or not with vitamin B3) on total phenolic content, activity of perdoxidase (POD) and polyphenol oxidase (PPO), and soluble sugar content of faba bean plants (*cv.* Giza 843) infected with *Rhizoctonia solani* under greenhouse conditions at 15 days after inoculation

Treatment	Total phenols (mg g^−1^ fresh weight)	POD (U g^−1^ fresh weight)	PPO (U g^−1^ fresh weight)	SSC (°Brix)
C	417.5 ± 12.3^m^	7.77 ± 0.3^l^	5.6 ± 0.4^j^	10.7 ± 1.5^f^
Rs	580.3 ± 10.5^k^	9.67 ± 0.7^j^	7.3 ± 0.6^h^	6.5 ± 0.3^g^
F + Rs	542.3 ± 11.2^l^	9.0 ± 0.6^k^	6.7 ± 0.5^i^	8.3 ± 0.8^g^
TH	629.6 ± 12.0^j^	12.9 ± 0.9^i^	8.9 ± 0.4^g^	17.0 ± 1.7^cd^
THB	696.3 ± 13.3^i^	13.5 ± 0.9^h^	9.3 ± 0.6^fg^	18.0 ± 1.6^bcd^
AMF	791.5 ± 13.7^h^	13.9 ± 0.7^h^	9.6 ± 0.8^f^	19.0 ± 1.5^bc^
TH + AMF	866.7 ± 14.6^g^	14.6 ± 0.6^g^	10.5 ± 0.5^e^	20.0 ± 1.9^b^
THB + AMF	960.4 ± 15.3^f^	15.9 ± 0.5^f^	11.7 ± 0.4^d^	23.0 ± 1.9^a^
TH + Rs	1090.3 18.9^e^	17.4 ± 0.7^e^	11.6 ± 0.7^d^	13.0 ± 1.6^e^
THB + Rs	1131.2 ± 20.3^d^	18.9 ± 0.5^d^	12.3 ± 0.9^c^	14.0 ± 1.5^e^
AMF + Rs	1200.1 ± 21.7^c^	19.6 ± 0.7^c^	12.9 ± 0.9^b^	16.0 ± 1.5^d^
TH + AMF + Rs	1286.0 ± 22.0^b^	21.3 ± 0.9^b^	13.4 ± 0.7^b^	18.0 ± 1.7^bcd^
THB + AMF + Rs	1402.3 ± 22.8^a^	23.7 ± 0.6^a^	14.6 ± 0.8^a^	20.0 ± 1.5^b^

Means within each column followed by different letter are significantly different, as determined by Duncan’s multiple range test at *P* ≤ 0.05. Where, C: untreated and not infected, Rs: untreated and infected, F + Rs: treated with the chemical fungicide and infected, TH: treated with *T. harzianum* HE24 and not infected, THB: treated with *T. harzianum* HE24/vitamin B3 and not infected, AMF: inoculated with AMF and not infected, TH + AMF: inoculated with AMF and treated with *T. harzianum* HE24 and not infected, THB + AMF: inoculated with AMF and treated with *T. harzianum* HE24/vitamin B3 and not infected, TH + Rs: treated with *T. harzianum* HE24 and infected, THB + Rs: treated with *T. harzianum* HE24/vitamin B3 and infected, AMF + Rs: inoculated with AMF and infected, TH + AMF + Rs: inoculated with AMF and treated with *T. harzianum* HE24 and infected, and TH + AMF + Rs: inoculated with AMF and treated with *T. harzianum* HE24/vitamin B3 and infected

### Effect of different treatments on mycorrhizal colonization

Mycorrhizal status in faba bean in response to the applied treatments are presented in Table 5. Microscopic observations of faba bean roots revealed all treatments which did not reciev AMF inoculum showed no mycorrhizal colonization. In contrast, all those inoculated with AMF showed varying degrees of mycorrhization. In this regard, the highest mycorrhization indexes were observed for the faba bean plants which were singly inoculated with AMF, recording colonization frequency (78.5%), colonization intensity (53.6%), and frequency of arbuscules (33.1%). No significant differences were recorded between faba bean plants only inoculated with AMF and those inoculated with AMF and *T. harzianum* HE24 (treated or not with vitamin B3). While, the lowest colonization level was observed for the faba bean which were inoculated with AMF and infected with Rs, recording colonization frequency (65.5%), colonization intensity (31.0%), and frequency of arbuscules (18.7%).

**Table 5:** Micorrhizal status of faba bean roots (*cv.* Giza 843) infected with *Rhizoctonia solani* in response to inoculation with arbuscular mycorrhizal fungi and/or *Trichoderma harzianum* HE24 (treated or not with vitamin B3) under greenhouse conditions at 30 days after inoculation

Treatment	Colonization frequency (%)	Colonization intensity (%)	Arbuscules frequency (%)
C	0^d^	0^d^	0^d^
Rs	0^d^	0^d^	0^d^
F + Rs	0^d^	0^d^	0^d^
TH	0^d^	0^d^	0^d^
THB	0^d^	0^d^	0^d^
AMF	78.5 ± 3.6^a^	53.6 ± 2.5^a^	33.1 ± 3.6^a^
TH + AMF	77.1 ± 2.8^a^	54.1 ± 2.7^a^	31.9 ± 2.4^a^
THB + AMF	78.3 ± 2.4^a^	54.3 ± 1.8^a^	31.5 ± 2.1^a^
TH + Rs	0^d^	0^d^	0^d^
THB + Rs	0^d^	0^d^	0^d^
AMF + Rs	65.5 ± 1.7^c^	31.0 ± 2.1^c^	18.7 ± 2.6^c^
TH + AMF + Rs	70.4 ± 2.5^b^	44.8 ± 2.2^b^	25.6 ± 1.8^b^
THB + AMF + Rs	71.6 ± 2.0^b^	45.3 ± 3.0^b^	26.0 ± 2.3^b^

Means within each column followed by different letter are significantly different, as determined by Duncan’s multiple range test at *P* ≤ 0.05. Where, C: untreated and not infected, Rs: untreated and infected, F + Rs: treated with the chemical fungicide and infected, TH: treated with *T. harzianum* HE24 and not infected, THB: treated with *T. harzianum* HE24/vitamin B3 and not infected, AMF: inoculated with AMF and not infected, TH + AMF: inoculated with AMF and treated with *T. harzianum* HE24 and not infected, THB + AMF: inoculated with AMF and treated with *T. harzianum* HE24/vitamin B3 and not infected, TH + Rs: treated with *T. harzianum* HE24 and infected, THB + Rs: treated with *T. harzianum* HE24/vitamin B3 and infected, AMF + Rs: inoculated with AMF and infected, TH + AMF + Rs: inoculated with AMF and treated with *T. harzianum* HE24 and infected, and TH + AMF + Rs: inoculated with AMF and treated with *T. harzianum* HE24/vitamin B3 and infected

### Effect of applying *T. harzianum* HE24 and/or AMF on total photosynthetic pigments

Table 6 shows effect of the tested treatments on photosynthetic pigments in faba bean leaves, including chl *a* and *b*, carotenoids. Results showed that Rs infection notably reduced all pigment levels, especially chl *a* recording 0.7 mg g^−1^ and total pigments (1.4 mg g^−1^), compared to the untreated and not infected plants (3.862 mg g^−1^). All applied treatments led to an increment in the total pigment, at varying degrees including the infected plants. The highest value was recorded for the faba bean plants treated with *T. harzianum* HE24 (treated with vitamin B3) and AMF recording 4.9 mg g^−1^, followed by the treatments TH + AMF and AMF alone recording 4.7 and 4.6 mg g^−1^, respectively.

**Table 6:** Effect of inoculation with arbuscular mycorrhizal fungi and/or *Trichoderma harzianum* HE24 (treated or not with vitamin B3) on content of the photosynthetic pigments (mg g^−1^ fresh weight) of faba bean leaves (*cv.* Giza 843) infected with *Rhizoctonia solani* under greenhouse conditions at 40 days after inoculation

Treatment	Chl *a*	Chl *b*	Carotenoids	Total pigments
C	1.785 ± 0.5^f^	0.594 ± 0.1^f^	0.521 ± 0.04^cde^	3.862 ± 0.4^g^
Rs	0.722 ± 0.3^g^	1.556 ± 0.2^bc^	0.066 ± 0.01^g^	1.382 ± 0.2^h^
F + Rs	1.823 ± 0.2^f^	1.417 ± 0.2^de^	0.510 ± 0.03^de^	3.759 ± 0.4^g^
TH	2.241 ± 0.3^e^	1.488 ± 0.1^bcd^	0.550 ± 0.05^bcd^	4.278 ± 0.5^e^
THB	2.325 ± 0.4^cde^	1.566 ± 0.2^abc^	0.560 ± 0.04^bcd^	4.452 ± 0.3^cd^
AMF	2.419 ± 0.5^b^	1.580 ± 0.1^ab^	0.580 ± 0.04^abc^	4.579 ± 0.4^bc^
TH + AMF	2.493 ± 0.3^b^	1.596 ± 0.2^ab^	0.590 ± 0.06^ab^	4.679 ± 0.4^b^
TH + AMF	2.653 ± 0.2^a^	1.699 ± 0.3^a^	0.627 ± 0.04^a^	4.979 ± 0.6^a^
TH + Rs	1.799 ± 0.2^f^	1.605 ± 0.1^ab^	0.427 ± 0.07^f^	3.832 ± 0.5^g^
THB + Rs	2.210 ± 0.5^e^	1.339 ± 0.2^e^	0.468 ± 0.08^ef^	4.018 ± 0.5^f^
AMF + Rs	2.272 ± 0.4^de^	1.437 ± 0.2^cde^	0.493 ± 0.05^e^	4.202 ± 0.6^e^
TH + AMF + Rs	2.296 ± 0.3^de^	1.509 ± 0.1^bcd^	0.562 ± 0.04^bcd^	4.367 ± 0.4^de^
THB + AMF + Rs	2.370 ± 0.4^cd^	1.699 ± 0.3^a^	0.583 ± 0.03^abc^	4.583 ± 0.5^bc^

Means within each column followed by different letter are significantly different, as determined by Duncan’s multiple range test at *P* ≤ 0.05. Where, C: untreated and not infected, Rs: untreated and infected, F + Rs: treated with the chemical fungicide and infected, TH: treated with *T. harzianum* HE24 and not infected, THB: treated with *T. harzianum* HE24/vitamin B3 and not infected, AMF: inoculated with AMF and not infected, TH + AMF: inoculated with AMF and treated with *T. harzianum* HE24 and not infected, THB + AMF: inoculated with AMF and treated with *T. harzianum* HE24/vitamin B3 and not infected, TH + Rs: treated with *T. harzianum* HE24 and infected, THB + Rs: treated with *T. harzianum* HE24/vitamin B3 and infected, AMF + Rs: inoculated with AMF and infected, TH + AMF + Rs: inoculated with AMF and treated with *T. harzianum* HE24 and infected, and TH + AMF + Rs: inoculated with AMF and treated with *T. harzianum* HE24/vitamin B3 and infected

### Effect of applying *T. harzianum* HE24 and/or AMF on faba bean growth

Table 7 shows effect of the tested treatments on faba bean growth parameters, including shoot height, root length, dry weights, branch count, and leaf area. Rs infection significantly reduced all parameters, with shoot dry weight dropping to 2.1 g and leaf area to 23.0 cm², comparing with the untreated not infected plants that recorded 4.8 g shoot dry weight and 55.0 cm² leaf area. All tested bioagents significantly enhanced the evaluated growth paameters, at varying degrees, even the infected plants. The TH treatment improved the plant growth, recording a shoot height of 66.7 cm and leaf area of 75.0 cm², while *T. harzianum* HE24**/**vitamin B3 was more effective in enhancing shoot dry weight (6.2 g) and leaf area (83.0 cm²). While fungicide increased growth relative to infected plants, it did not reach the healthy control levels. The THB + AMF treatment resulted in the highest shoot dry weight (7.9 g), shoot height (74.3 cm), and leaf area (107.3 cm²). The combination of THB + AMF + Rs was also effective, achieving a shoot height of 68.0 cm and leaf area of 98.8 cm² under Rs infection.

**Table 7:** Effect of inoculation with arbuscular mycorrhizal fungi and/or *Trichoderma harzianum* HE24 (treated or not with vitamin B3) on growth of faba bean plants (*cv.* Giza 843) infected with *Rhizoctonia solani* under greenhouse conditions at 50 days after inoculation

Treatment	Shoot height (cm)	Root length (cm)	Shoot dry weight (g)	Root dry weight (g)	Number of branches	Leaf area (cm^2^)
C	38.2 ± 4.1^f^	7.1 ± 0.9^j^	4.8 ± 0.7^e^	1.0 ± 0.4^e^	1.67 ± 0.7^cd^	55.0 ± 4.0^h^
Rs	56.7 ± 5.2^de^	12.5 ± 1.1^h^	2.1 ± 0.9^g^	0.4 ± 0.1^f^	1.0 ± 0.2^d^	23.0 ± 3.1^j^
F + Rs	51.7 ± 4.7^e^	10.0 ± 1.3^i^	4.1 ± 0.9^f^	0.9 ± 0.2^e^	1.67 ± 0.4^cd^	50.9 ± 4.1^i^
TH	66.7 ± 6.1^bc^	16.6 ± 1.9^d^	5.5 ± 1.1^d^	1.1 ± 0.5^de^	2.0 ± 0.3^bcd^	75.0 ± 4.7^f^
THB	67.7 ± 4.0^abc^	17.4 ± 1.1^cd^	6.2 ± 1.1^c^	1.4 ± 0.7^cd^	2.7 ± 0.7^abc^	83.1 ± 5.0^d^
AMF	67.3 ± 3.9^abc^	17.9 ± 1.5^bc^	7.0 ± 0.9^b^	1.6 ± 0.7^bc^	3.0 ± 1.1^ab^	93.4 ± 6.2^c^
TH + AMF	72.3 ± 4.5^ab^	18.7 ± 1.7^ab^	7.2 ± 0.7^b^	1.8 ± 1.3^ab^	3.3 ± 1.7^a^	104.2 ± 5.7^a^
THB + AMF	74.3 ± 5.8^a^	19.5 ± 1.5^a^	7.9 ± 0.6^a^	2.0 ± 1.6^a^	3.3 ± 1.5^a^	107.3 ± 4.9^a^
TH + Rs	61.3 ± 4.1^cd^	13.3 ± 1.3^gh^	5.0 ± 0.5^e^	0.9 ± 0.5^e^	1.7 ± 0.8^cd^	67.4 ± 4.3^g^
THB + Rs	62.3 ± 4.8^cd^	14.2 ± 1.1^fg^	5.4 ± 0.9^d^	1.1 ± 0.4^de^	2.0 ± 1.0^bcd^	79.1 ± 4.4^e^
AMF + Rs	63.5 ± 5.5^c^	14.6 ± 1.1^ef^	6.2 ± 0.7^c^	1.4 ± 0.5^cd^	2.7 ± 0.7^abc^	91.0 ± 4.9^c^
TH + AMF + Rs	65.0 ± 4.0^bc^	15.5 ± 1.5^e^	6.8 ± 0.6^b^	1.5 ± 0.6^bc^	3.0 ± 0.6^ab^	94.3 ± 4.8^c^
THB + AMF + Rs	68.0 ± 4.7^abc^	15.2 ± 1.6^ef^	6.9 ± 0.8^b^	1.7 ± 0.9^abc^	3.3 ± 0.9^a^	98.8 ± 45.1^b^

Means within each column followed by different letter are significantly different, as determined by Duncan’s multiple range test at *P* ≤ 0.05. Where, C: untreated and not infected, Rs: untreated and infected, F + Rs: treated with the chemical fungicide and infected, TH: treated with *T. harzianum* HE24 and not infected, THB: treated with *T. harzianum* HE24/vitamin B3 and not infected, AMF: inoculated with AMF and not infected, TH + AMF: inoculated with AMF and treated with *T. harzianum* HE24 and not infected, THB + AMF: inoculated with AMF and treated with *T. harzianum* HE24/vitamin B3 and not infected, TH + Rs: treated with *T. harzianum* HE24 and infected, THB + Rs: treated with *T. harzianum* HE24/vitamin B3 and infected, AMF + Rs: inoculated with AMF and infected, TH + AMF + Rs: inoculated with AMF and treated with *T. harzianum* HE24 and infected, and TH + AMF + Rs: inoculated with AMF and treated with *T. harzianum* HE24/vitamin B3 and infected

### Effect of applying *T. harzianum* HE24 and/or AMF on faba bean yield

Table 8 indicates effects of various treatments on yield parameters of faba bean plants, specifically pod weight, seed count per pod, pod length, and pod width. The infected plants showed marked reductions across all evaluated yield parameters recording pod weight (4.0 g), length (5.0 cm), and width (1.2 cm), and seed counts (1.7), compared to the untreated control plants which recorded pod weight (7.4 g), length (6.7 cm), and width (1.6 cm), and seed counts (2.3). While applying the tested treatments, alone or in combinations, enhanced the growth in the infected plants. The THB + AMF treatment significantly enhanced the yield achieving the highest pod weight (14.7 g), length (12.2 cm), and width (2.0 cm), and seed counts (4.0).

**Table 8:** Effect of inoculation with arbuscular mycorrhizal fungi and/or *Trichoderma harzianum* HE24 (treated or not with vitamin B3) on yield of faba bean plants (*cv.* Giza 843) infected with *Rhizoctonia solani* under greenhouse conditions

Treatment	Weight of pod (g)	Number of seeds per pod	Length of pod (cm)	Width of pod (cm)
C	7.4 ± 0.8^e^	2.3 ± 0.4^bc^	6.7 ± 0.7^f^	1.6 ± 0.2^c^
Rs	4.0 ± 0.3^g^	1.7 ± 0.3^c^	5.0 ± 0.5^g^	1.2 ± 0.2^d^
F + Rs	5.3 ± 0.5^f^	2.3 ± 0.2^bc^	6.1 ± 0.4^f^	1.6 ± 0.4^bc^
TH	11.0 ± 1.1^bc^	2.3 ± 0.3^bc^	9.0 ± 0.6^cde^	1.8 ± 0.3^abc^
THB	11.4 ± 1.1^bc^	3.0 ± 0.7^abc^	10.0 ± 0.8^bcd^	1.9 ± 0.2^a^
AMF	11.2 ± 1.2^bc^	3.3 ± 0.8^ab^	10.0 ± 0.9^bcd^	2.0 ± 0.3^a^
TH + AMF	13.9 ± 1.5^a^	3.3 ± 0.6^ab^	10.7 ± 0.8^bc^	2.0 ± 0.4^a^
THB + AMF	14.7 ± 1.6^a^	4.0 ± 0.5^a^	12.2 ± 1.1^a^	2.0 ± 0.2^a^
TH + Rs	8.08 ± 0.8^e^	3.0 ± 0.5^abc^	8.4 ± 0.5^e^	1.7 ± 0.1^abc^
THB + Rs	9.6 ± 0.6^d^	3.0 ± 0.6^abc^	8.5 ± 0.4^de^	1.8 ± 0.3^abc^
AMF + Rs	10.5 ± 0.8^c^	3.3 ± 0.5^ab^	9.0 ± 0.6^cde^	1.9 ± 0.2^ab^
TH + AMF + Rs	11.6 ± 1.0^bc^	3.0 ± 0.4^abc^	10.3 ± 0.9^bc^	1.8 ± 0.2^abc^
THB + AMF + Rs	11.8 ± 0.9^b^	3.3 ± 0.2^ab^	10.3 ± 0.8^bc^	1.9 ± 0.3^ab^

Means within each column followed by different letter are significantly different, as determined by Duncan’s multiple range test at *P* ≤ 0.05. Where, C: untreated and not infected, Rs: untreated and infected, F + Rs: treated with the chemical fungicide and infected, TH: treated with *T. harzianum* HE24 and not infected, THB: treated with *T. harzianum* HE24/vitamin B3 and not infected, AMF: inoculated with AMF and not infected, TH + AMF: inoculated with AMF and treated with *T. harzianum* HE24 and not infected, THB + AMF: inoculated with AMF and treated with *T. harzianum* HE24/vitamin B3 and not infected, TH + Rs: treated with *T. harzianum* HE24 and infected, THB + Rs: treated with *T. harzianum* HE24/vitamin B3 and infected, AMF + Rs: inoculated with AMF and infected, TH + AMF + Rs: inoculated with AMF and treated with *T. harzianum* HE24 and infected, and TH + AMF + Rs: inoculated with AMF and treated with *T. harzianum* HE24/vitamin B3 and infected

## Discussion

*Rhizoctonia solani*, a soil-borne fungal pathogen, poses a significant threat to faba bean production by causing root rot. Due to its wide host range and the high genetic variability within its populations, conventional chemical control methods are often of limited effectiveness and may contribute to soil degradation and environmental concerns [7, 8, 22]. In the present study, *T. harzianum* HE24 (with or without vitamin B3 treatment) and/or AMF were evaluated for their biocontrol efficacy against Rs as a safer, eco-friendly alternative to chemical fungicides. *Trichoderma harzianum* HE24 exhibited significant antagonistic potential by reducing the growth of Rs *in vitro*. This inhibitory effect may be attributed to its ability to produce antifungal metabolites and/or hydrolytic enzymes such as chitinase and glucanase, which are capable of degrading the fungal cell wall of Rs [2, 23]. In a previous study, production of a set of antifungal metabolites by *T. harzianum* HE24 was reported via GC-MS analysis [24]. The produced antifungal compounds included 2,3-butanediol, 9-octadecenamide, carvacrol, decane, 2,4,6-trimethyl-, phenylethyl alcohol, and acetic acid. The recorded antifungal activity of *T. harzianum* HE24 can be attributed to these active metabolites. This result was supported by SEM observations, which revealed significant morphological alterations in mycelia of Rs in response to metabolites produced by *T. harzianum* HE24. These alterations included distortion and disintegration of its mycelia along with coiling and penetration by mycelia of *T. harzianum* HE24 which indicated its mycoparasitic behavior. These findings suggest that multiple modes of action contribute to the antagonistic activity of *T. harzianum* HE24. This result is in the same line with the findings of Natey et al. [25] who reported similar mycoparasitic interactions of *T. afroharzianum* B3R12 against the onion white rot pathogen *Stromatinia cepivora.* Various antagonistic mechanisms of *Trichoderma* spp. have been described against various fungal pathogens including competition for space and/or nutrients, antibiosis by producing plethora of volatile and/or nonvolatile antifungal metabolites such as pyrones, butanolides, volatile terpenes, peptaibols, gliotoxin, and gliovirin [2, 4]. In addition, *Trichoderma* spp. can directly parasitize fungal pathogens, leading to structural damage such as hyphal distortion, vacuolation, cell wall disintegration, enzymatic suppression, and ultimately, cell lysis [26].

Furthermore, the results showed that vitamin B3 enhanced antifungal activity of *T. harzianum* HE24 by stimulating metabolic pathways involved in the production of hydrolytic enzymes and antifungal secondary metabolites. This result aligns with that of Yousef et al. [4], who reported that supplementing *Trichoderma* culture media with certain chemical inducers such as potassium tartrate, micronutrient mixtures, and thiamine, enhanced the antagonistic activity of *T. harzianum* against Rs. Vitamin B3 has also been shown to enhance microbial metabolism, stimulate the production of secondary metabolites, and improve the overall growth and activity of *Chlorella vulgaris* [27]. Therefore, the incorporation of vitamin B3 in *Trichoderma* applications may represent a promising strategy to boost the effectiveness of biological control against fungal diseases.

In the greenhouse experiment, the biocontrol efficacy of *T. harzianum* HE24 (with or without vitamin B3) and/or AMF was evaluated against Rhizoctonia root rot of faba bean plants. A significant reduction in the disease severity was obtained in response to application of *T. harzianum* HE24 (with vitamin B3) and AMF. The additive interaction between *T. harzianum* HE24 and AMF likely enhanced disease suppression by improving nutrient availability and promoting extensive root colonization, consistent with findings from previous studies [2, 28]. The obtained results from the greenhouse experiment showed that this combined treatment triggered transcriptional upregulation of key defense-related genes *CHI II*,* PAL1*, and *HQT* in faba bean plants challenged with Rs. *CHI II* encodes for chitinase, an antifungal enzyme that hydrolyzes chitin, a major structural component of fungal cell walls. Its overexpression is recognized as a critical defense mechanism in plants under fungal attack [29]. Triggering expression of *CHI II* due to colonization the plant roots with AMF has been widely reported across many plant species [8]. *PAL1* plays an important role in plant defense, as it is central to the polyphenols biosynthesis pathway, which include production of a plethora of fungitoxic metabolites such as flavonoids, flavones, flavonols, chlorogenic acid, and lignins [30]. These polyphenolic compounds represent the main defense lines that suppress the pathogen development and transfer from cell to cell in the plant tissue [3]. *HQT* is a main gene in the biosynthesis of chlorogenic acid pathway. Induction of *HQT* expression has been reported due to colonization with AMF [3]. Chlorogenic acid, which accumulates in plant tissues in response to pathogenic fungi, has a strong antifungal activity via induction of fungal cell lysis and membrane permeabilization of the spores [31].

This study demonstrated that the application of *T. harzianum* HE24 (with or without vitamin B3) and/or AMF effectively enhances the biochemical defense responses in faba bean plants under Rs infection. While infection by the pathogen alone induced accumulation of the phenolic compounds and enhanced activity of POD and PPO as a part of the natural stress responses, the application of these biological agents additively amplified these responses in an additive manner—surpassing the levels observed in untreated infected plants. This finding is in agreement with that of the previous studies [32, 33], which reported that application of *Trichoderma* spp. boosted phenolic biosynthesis pathways to fortify plants against pathogens. POD and PPO enzymes play essential roles in lignin synthesis and ROS scavenging which are critical for structural defense [34]. Phenolic compounds are crucial for reinforcing cell walls and inhibiting pathogen invasion [35]. Additionally, Eid et al. [36] explained the role of AMF and environmentally biochemicals in enhancing the nutritional status of *Helianthus tuberosus* and inducing its resistance against *Sclerotium rolfsii.*

AMF similarly enhanced PPO and SSC levels, indicating their roles in optimizing nutrient availability, which supports energy production and strengthens plant defenses. Rashad et al. [3] showed that AMF facilitates root colonization and nutrient uptake, enhancing plant resistance under pathogen stress. The combined treatment of THB + AMF yielded the highest levels of phenolics, POD, PPO, and SSC, indicating a powerful additive effect. This finding is in consistence with that of El-Sharkawy et al. [37, 38] who reported that combining biocontrol agents and metabolic enhancers optimizes plant metabolic and defense responses under pathogen stress. AMF have been known to play many beneficial roles in their hosts including enhancing the water and nutrients uptake from the soil, inducing different defense responses against biotic stresses, and triggering the plant tolerance to abiotic stresses such as drought and salinity [39].

This study highlights the potential of the tested treatments in mitigating the negative effects of Rs on faba bean plants by preserving and enhancing photosynthetic pigment levels. Chlorophyll a, b, and carotenoids are critical components of the photosynthetic apparatus, and their reduction due to pathogen infection can severely affect plant growth and yield. This result aligns with recent studies showing that *T. harzianum* promotes plant growth by enhancing photosynthetic pigment production. El-Sharkawy et al. [33] reported that *T. harzianum* boosts chlorophyll content and stress tolerance in plants by activating plant defense mechanisms and improving nutrient uptake. AMF’s role in improving photosynthetic pigments is well-documented. Studies by El-Gazzar et al. [22] and El-Sharkawy et al. [2] showed that AMF have a great role in enhancing plant nutrition, particularly phosphorus uptake, which is crucial for the chlorophyll biosynthesis [40–42].

Rs infection significantly reduced yield parameters like pod weight, pod length, and seed number. However, treatments with *T. harzianum* HE24 and AMF, especially when supplemented with vitamin B3, substantially improved yield, demonstrating their efficacy in mitigating pathogen effects. *Trichoderma* spp. have been shown to enhance plant growth and yield under stress by promoting nutrient solubilization, root growth, and systemic resistance, as noted by Asghar et al. [43] and El-Sharkawy et al. [2]. AMF are well-known for improving nutrient uptake, especially phosphorus, and enhancing plant tolerance to both biotic and abiotic stresses [6, 28]. By forming symbiotic relationships with the plant roots, AMF also modulate root exudates, which may facilitate better growth of *Trichoderma* in the rhizosphere. This additive effect can explain the improved biocontrol efficacy observed in the dual treatments including *Trichoderma* HE24 and AMF [44].

Based on the obtained results, we can conclude that using a combination of *T. harzianum* HE24, with vitamin B3 supplementation, and AMF represents a promising eco-friendly strategy warranting further field validation for managing *R. solani*-induced root rot in faba bean. This biocontrol approach not only suppressed the pathogen growth but also enhanced faba bean growth and resistance. Future research is necessary to focus on optimizing application methods and dosages across diverse environmental conditions and soil types to maximize efficacy. Expanding field trials and exploring potential applications for other crops may further support sustainable agricultural practices and broader crop protection solutions.

## Data Availability

All authors declare that the obtained data in this study will be available upon reasonable re-quest.
